# Identification of a Highly Conserved H1 Subtype-Specific Epitope with Diagnostic Potential in the Hemagglutinin Protein of Influenza A Virus

**DOI:** 10.1371/journal.pone.0023374

**Published:** 2011-08-19

**Authors:** Rongmao Zhao, Shujuan Cui, Li Guo, Chao Wu, Richard Gonzalez, Gláucia Paranhos-Baccalà, Guy Vernet, Jianwei Wang, Tao Hung

**Affiliations:** 1 State Key Laboratory of Molecular Virology and Genetic Engineering, and Christophe Mérieux Laboratory, Institute of Pathogen Biology, Peking Union Medical College and Chinese Academy of Medical Sciences, Beijing, China; 2 Fondation Mérieux, Lyon, France; New York Blood Center, United States of America

## Abstract

Subtype specificity of influenza A virus (IAV) is determined by its two surface glycoproteins, hemagglutinin (HA) and neuraminidase (NA). For HA, 16 distinct subtypes (H1–H16) exist, while nine exist for NA. The epidemic strains of H1N1 IAV change frequently and cause annual seasonal epidemics as well as occasional pandemics, such as the notorious 1918 influenza pandemic. The recent introduction of pandemic A/H1N1 IAV (H1N1pdm virus) into humans re-emphasizes the public health concern about H1N1 IAV. Several studies have identified conserved epitopes within specific HA subtypes that can be used for diagnostics. However, immune specific epitopes in H1N1 IAV have not been completely assessed. In this study, linear epitopes on the H1N1pdm viral HA protein were identified by peptide scanning using libraries of overlapping peptides against convalescent sera from H1N1pdm patients. One epitope, P5 (aa 58–72) was found to be immunodominant in patients and to evoke high titer antibodies in mice. Multiple sequence alignments and *in silico* coverage analysis showed that this epitope is highly conserved in influenza H1 HA [with a coverage of 91.6% (9,860/10,767)] and almost completely absent in other subtypes [with a coverage of 3.3% (792/23,895)]. This previously unidentified linear epitope is located outside the five well-recognized antigenic sites in HA. A peptide ELISA method based on this epitope was developed and showed high correlation (χ^2^ = 51.81, P<0.01, Pearson correlation coefficient R = 0.741) with a hemagglutination inhibition test. The highly conserved H1 subtype-specific immunodominant epitope may form the basis for developing novel assays for sero-diagnosis and active surveillance against H1N1 IAVs.

## Introduction

Influenza A viruses (IAVs), members of the *Orthomyxoviridae* family, are highly contagious to a variety of avian and mammalian species. IAVs cause seasonal influenza epidemics annually and recurring pandemics with severe consequences for public health and global economy [1], [2]. At least three IAV-pandemics emerged in the last century (1918 A/H1N1, 1957 A/H2N2, and 1968 A/H3N2). The 1918 Spanish flu was the most serious influenza pandemic that killed over 50 million people worldwide [3]. The latter two pandemics, although mild compared to the 1918 incidence, resulted in significant mortality, with close to 2 million and 1 million deaths, respectively [4]. The latest pandemic influenza, and newest global health challenge, occurred in 2009 due to the emergence of an A/H1N1 pandemic IAV (H1N1pdm virus). The H1N1pdm virus has been detected in more than 214 countries and territories and has caused 18,389 deaths as of July 30, 2010 [5].

The viral genome of IAV consists of eight single-stranded negative sense RNA segments that encode at least 11 viral proteins, including two surface glycoproteins, hemagglutinin (HA) and neuraminidase (NA) [6]. Based on the antigenic properties of HA and NA, IAVs have been classified into 16 HA subtypes and 9 NA subtypes [7]. All 16 HA subtypes have been identified in avian species, while only 6 HA subtypes (H1, H2, H3, H5, H7 and H9) are known to infect human beings [8], [9], [10]. H1, H2 and H3 subtypes have caused pandemics, while H1 and H3 also dominate seasonal epidemics together with influenza B virus.

HA, encoded by segment 4 of the IAV genome, is a glycoprotein of approximate 560 amino acid. The biologically active HA is a homologous trimeric molecule that is attached to the virion membrane through its carboxy terminus [11]. HA plays a critical role in the pathogenesis of IAVs. HA mediates IAVs' binding to the cellular receptor N-acetylneuraminic (sialic) acid as well as the subsequent membrane fusion process [12]. HA also stimulates host protective immunities, specifically the production of neutralizing antibodies. The generation of anti-HA neutralizing antibodies has been the major target for influenza vaccine development [11], [13]. Due to its specificity in immune response, HA is also an important target for IAV subtyping using immunoassays [7], [14].

Active serological surveillance for viral antibodies is of great importance for influenza control and prevention. Several IAV subtype-specific serological tests have been developed. At present, subtyping of IAV mainly relies on a hemagglutination inhibition (HI) test using HA and NA subtype-specific reference sera [15]. However, there are a number of drawbacks to HI testing. This assay is 1) relatively laborious; 2) low in sensitivity; 3) requires preparation of antigen from viable viruses which are potentially hazardous and 4) contains low signal to noise ratio, e.g. the assay exhibits inter-variability and subtype cross-reactivity [16], [17]. Moreover, the HI test can be confounded by steric hindrance from NA antibodies, leading to nonspecific inhibition and misidentification [18].

Microneutralizing test is an alternative method to type and subtype influenza viruses. However, due to the needs of cell culture process, this method is labor-intensive and requires biological safety containments (particularly for high pathogenic strains). As such, it is not suitable for large scale investigations [19], [20]. Recently, subtyping of IAV antibodies using different categories of ELISA assays have also been reported [16], [17], [21]. However, present ELISA assays mainly rely on an HA antigen, which can lead to nonspecific detection to some extent due to the possible cross-reaction of different subtypes [22], [23].

Virus-derived epitopes are useful tools to accurately evaluate immune response and to differentiate which responses are specific or due to cross-reactivity [24], [25], [26]. Several studies have reported the existence of HA subtype-specific as well as inter subtype-conserved epitopes [27], [28], [29]. ELISA assays based on epitopes that are highly conserved and specific for one certain HA subtype will be useful for rapid and simple subtyping of IAVs. Such epitopes in IAVs have not been fully addressed although many studies have been performed. In the present study, we report the successful identification of a new epitope, which is highly conserved among the majority of IAV strains of H1 subtype. Moreover, we developed an ELISA assay for H1 antibody subtyping based on this epitope. Results derived from this new assay correlate with results obtained through the use of HI test.

## Results

### Identification of immunodominant epitopes in the HA protein of H1N1pdm virus

**Figure 1:**
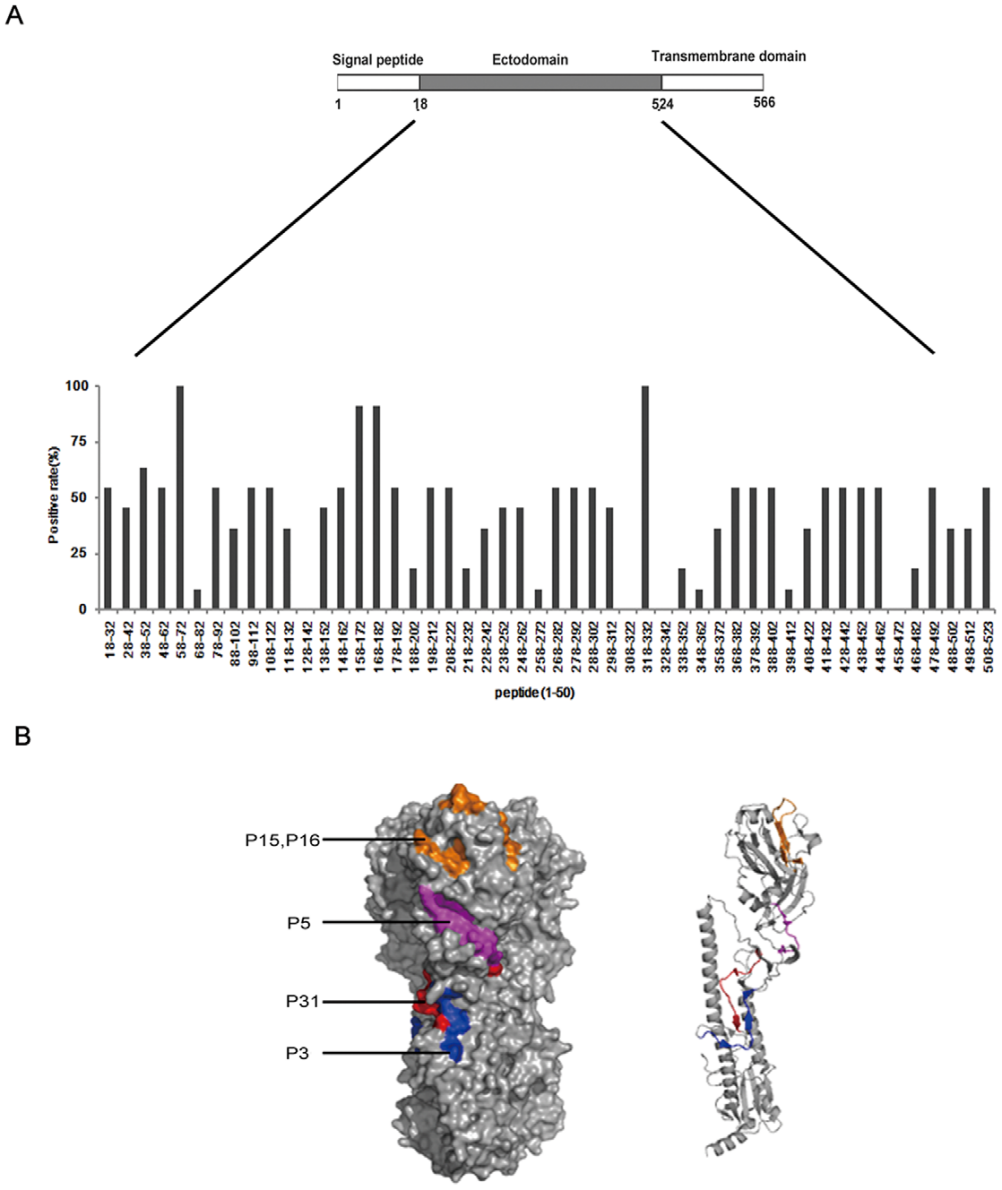
Identification of immunodominant epitopes in the HA protein of H1N1pdm influenza virus by peptide scanning analysis. (A) A set of 50 peptides that cover the ectodomain of the HA protein of A/California/04/2009 strain were used to coat 96-well microplate. Sera (1∶200 dilution) from 11 H1N1pdm patients and 10 healthy donors were screened for the presence of antibodies to the 50 peptides using ELISA. The positive rate of H1N1pdm sera for each peptide was calculated and plotted. (B) The identified peptides displayed in stereo view. The HA trimer surface view of H1N1pdm influenza virus (PDB ID:3LZG) is shown on the left and colored to illustrate the five immunodominant peptides. From most membrane distal to proximal: P15 and P16 (orange, residues 158–182), P5 (magenta, residues 58–73), P31 (red, residues 318–332), P3 (blue, residues 38–52). The HA monomer cartoon view is shown on the right and the same coloring scheme applies to the structure on the left.

To identify the immunodominant epitopes in the HA protein, a peptide scanning assay was performed. A set of 50 peptides with five residues overlapping with the adjacent peptides spanning the ectodomain sequences of the HA protein of the H1N1pdm virus strain A/California/04/2009 were synthesized. The binding between these peptides and the convalescent serum samples from 11 H1N1pdm patients were examined by ELISA using these peptides as coating antigens. Five of these peptides (P3, P5, P15, P16 and P31) were found to react well with the sera tested. These peptides corresponded to the sequences of amino acid (aa) residues 38–52, 58–72, 158–172, 168–182, and 318–332 in the HA molecule, respectively (Fig. 1A and Table 1). Among them, the P3 peptide reacted with 54.5% (6/11) of the sera, the P15 and P16 peptides reacted with 81.8% (9/11) of the sera, while the P5 and P31 peptides reacted with 100% (11/11) of the sera. These data indicated that these peptides may contain H1N1pdm virus B cell epitopes.

**Table 1:** Sequences of the peptides conjugated with KLH carrier for animal immunization.

Designation	Positions#	Sequences
P3-KLH	38–52	EKNVTVTHSVNLLED-KLH
P5-KKC-KLH	58–72	LCKLRGVAPLHLGKC-[Acp]-KKC-KLH*
P6-KLH	68–82	HLGKCNIAGWILGNP-KLH
P15-KKC-KLH	158–172	AKSFYKNLIWLVKKG-[Acp]-KKC-KLH
P16-KKC-KLH	168–182	LVKKGNSYPKLSKSY-[Acp]-KKC-KLH
P30-KLH	308–322	PFQNIHPITIGKCPK-KLH
P31-KKC-KLH	318–332	GKCPKYVKSTKLRLA-[Acp]-KKC-KLH

# Indicated as the position corresponding to the HA protein of pandemic A/H1N1 2009 influenza virus strain A/California/04/2009.

*Acp: coupled with 6-aminocaproic acid.

To visualize the location of the peptides on the HA protein, we mapped the peptides on the crystal model of this protein (Fig. 1B). The various colors in Figure 1B represent the different peptides. Although P3 (residues 38–52, indicated by blue) and P31 (residues 318–332, indicated by red) are parts of HA1 in primary sequence, they are located in the middle of helix A and B in the trimeric structure and are partially surface exposed. P5 (residues 58–72, indicated by magenta) seems to be a dispatch that links the stem region and the globular region and is fully surface exposed (Fig. 1B). P15 and P16 (residues 158–172 and 168–182, indicated by orange) are located in the receptor binding domain [11].

### Immunogenicity of immunodominant peptides

**Figure 2:**
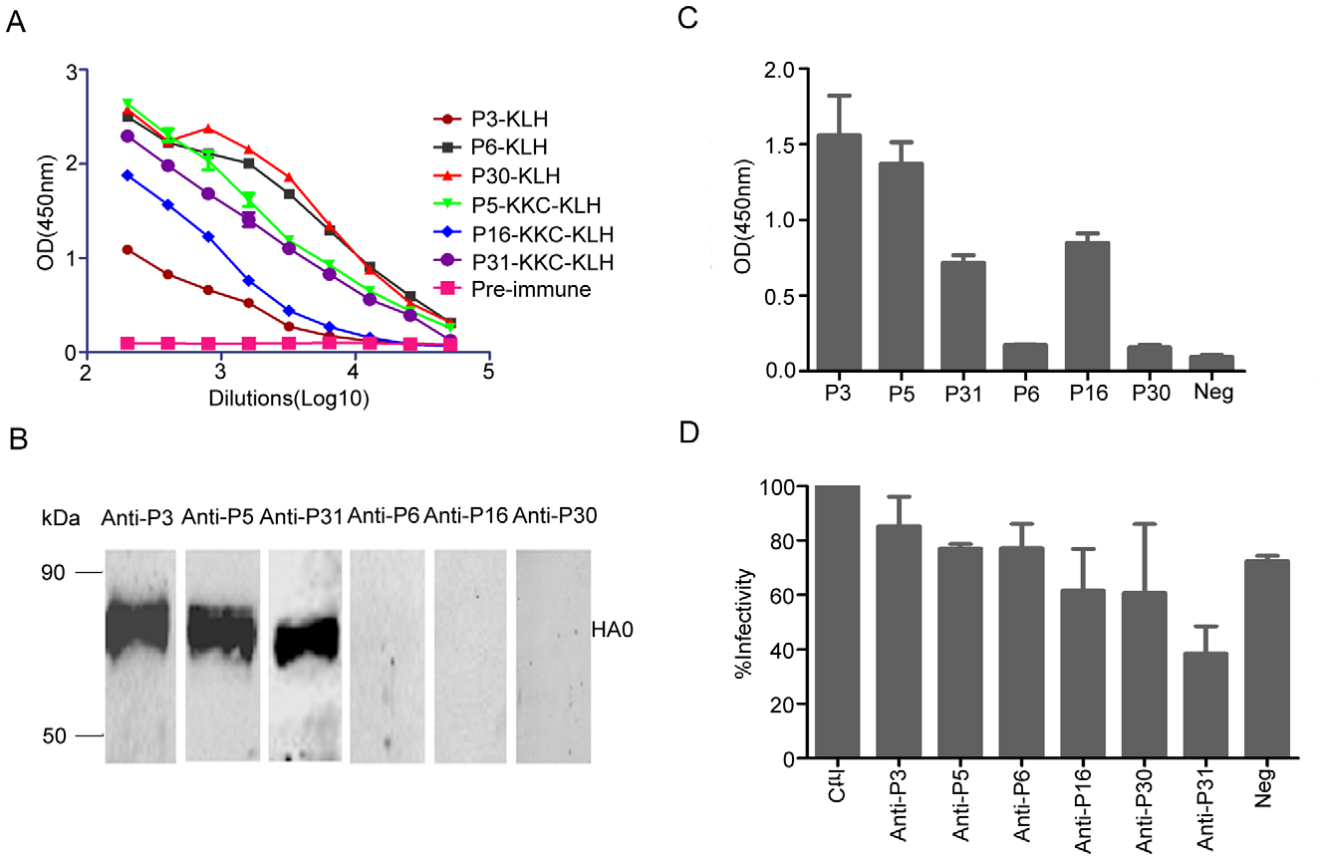
Immunogenicity of immunodominant peptides. (A) Titration of IgG antibody against peptides derived from the H1N1pdm virus. The titers of murine sera were determined as a series of two-fold dilutions by ELISA. (B and C) The reactivities of anti-peptide sera (1∶400 dilution) with the purified HA0 protein of the H1N1pdm virus were analyzed by Western blot (B) and ELISA (C). For the ELISA assay, the OD_450 nm_ values are expressed as mean ± SD. (D) Neutralization activity of anti-peptide sera. HA-pseudotype neutralization tests were performed to determine the neutralizing activity of the anti-sera derived from immunization with the peptides P3, P5, P6, P16, P30, and P31 in mice. The percentage of infectivity compared to that of negative serum (Ctrl) was calculated. Serum that induced a 90% reduction of infectivity was considered positive. Data were from at least duplicate testing of serum samples.

To confirm the immunogenicity of these peptides *in vivo*, we analyzed sera derived from peptide-immunized mice. The five positive peptides and two control peptides (P6 and P30) were coupled with keyhole limpet hemocyanin (KLH) and were used to immunize BALB/c mice (Table 1). The antisera were collected five days after the third immunization and titrated by ELISA using corresponding peptide as a coating antigen. Our results showed that all of the peptide conjugates except P15 induced potent antibody titers. The endpoint titers of antisera in ELISA from mice immunized with P3, P5, P6, P16, P30, and P31 peptides were 1∶6,400, 1∶51,200, 1∶51,200, 1∶12,800, 1∶51,200, and 1∶25,600, respectively (Fig. 2A). These data indicate that most of the positive peptides elicite humoral immunity and are highly immunogenic in mice.

To confirm that these antibodies can recognize the HA antigen, the reactivity of the anti-peptide sera were evaluated by Western blot and ELISA against the purified HA0 protein of H1N1pdm virus. Our data demonstrate that sera against P3, P5, and P31 but not those against P6 and P30 (controls) react to the HA0 protein (Fig. 2B and 2C). The anti-P16 sera did not react to the HA0 protein, although it exhibited a high ELISA reactivity to the HA0 protein (Fig. 2B and 2C). Taken together, our results demonstrate that P3, P5, and P31 peptides contain dominant epitopes of H1N1pdm virus. We then characterized these three peptides in the following studies.

To determine if the epitopes identified in this study can stimulate neutralizing antibodies, a HA-pseudotype neutralization test was performed against the anti-peptide sera using the H1N1pdm pseudotyped lentivirus. None of the sera against P3, P5, P16, and P31 could efficiently inhibit (90% inhibition [30]) the entry of H1N1pdm HA pseudotypes (Figure 2D), indicating that these epitopes do not contain neutralizing activity.

### Specificity of the identified epitopes

**Figure 3:**
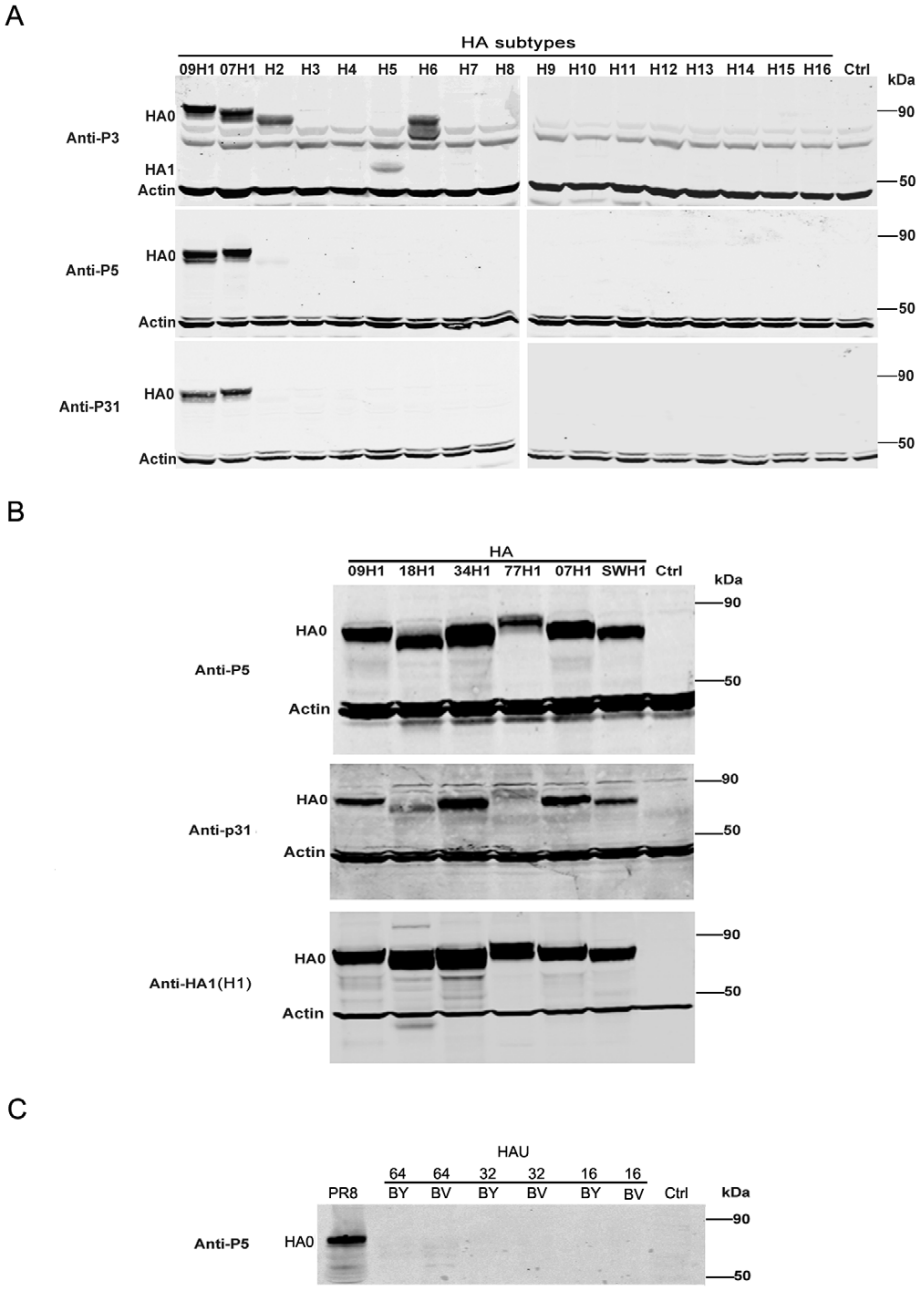
Specificity of antibodies induced by peptide conjugates. (A) Reactivities between antibodies against P3, P5 and P31 and H1–H16 HA proteins. Western blot analysis was performed using lysates from 293T cells transfected with the recombinant pCAGGS plasmids expressing H1–H16 HA proteins. For H1 subtype HA, HA proteins from a seasonal epidemic H1N1 strain A/Brisbane/59/2007 and an H1N1pdm strain (A/California/04/2009) were both tested. (B) Reactivities between antibodies against P3, P5 and P31 and HA proteins of H1 subtype strains. Western blot analysis was performed on 293T cell lysates expressing recombinant HA proteins from five human H1N1 strains isolated in different years (1918, 1934, 1977, 2007, 2009) and a swine H1N1 strain. 293T cells transfected with an empty vector was used as a control (Ctrl). β-actin was used as a loading control. For the backgrounds of various subtype IAV strains, see Fig. 4. (C) Reactivities between anti-P5 antibody and HA proteins of influenza B virus strains. Western blot analysis was performed using different hemagglutinating units (HAU) of influenza B virus strains B/hubeiwujiagang/158/2009 (BY) and B/heilongjianghulan/116/2010 (BV). An influenza A virus strain A/PR8/34 (H1N1) (PR8) was used as positive control. MDCK cells were used as a negative control (Ctrl).

Western blot analysis was used to determine the specificity of the epitopes present in the peptides P3, P5, and P31. The H1–H16 recombinant HA proteins were obtained by transient expressions of corresponding genes by the pCAGGS vector in 293T cells. The lysates of these cells were used to examine the specificity of antibodies elicited by peptide-conjugates. As shown in Fig. 3A, the anti-P3 serum reacted with H1 (including 07H1 and 09H1 viruses), H2, H5, and H6 HA proteins, while anti-P5 and anti-P31 sera only reacted with the H1 HA proteins. These findings indicated that P5 and P31 may contain H1-subtype specific epitopes.

To evaluate the subtype-specificity of epitopes in P5 and P31 further, additional HA proteins of three epidemic human strains from different years (1918, 1934 and 1977) as well as a swine strain were expressed by pCAGGS vector in 293T cells. The reactivity of anti-P5 and P31 sera with the cell lysates was determined by Western blot analysis. Our results showed that the anti-P5 serum strongly reacted with all of the six H1 HA proteins in a manner similar to an antibody against the HA1 of H1N1pdm virus (Fig. 3B). We found that the anti-P31 sera reactivity was weak against HA proteins from the 1918 and 1977 virus strains (Fig. 3B). These data indicated that the epitopes in P5 and P31 peptides are relatively conserved among H1-subtype IAVs though these viruses have circulated in the world for almost a century.

To test if anti-P5 sera cross-react with influenza type B virus, the reactivity of anti-P5 sera with two representative influenza type B virus strains (B/hubeiwujiagang/158/2009,Yamagata lineage and B/heilongjianghulan/116/2010, Victoria lineage) and an influenza type A virus strain (A/H1N1/PR8/34) was examined by Western blot analysis. The results showed that anti-P5 serum reacted well with A/H1N1/PR8/34 virus but not with influenza type B virus strains (Fig. 3C), further confirming the specificity of the epitope in P5.

### Conservation analysis

**Figure 4:**
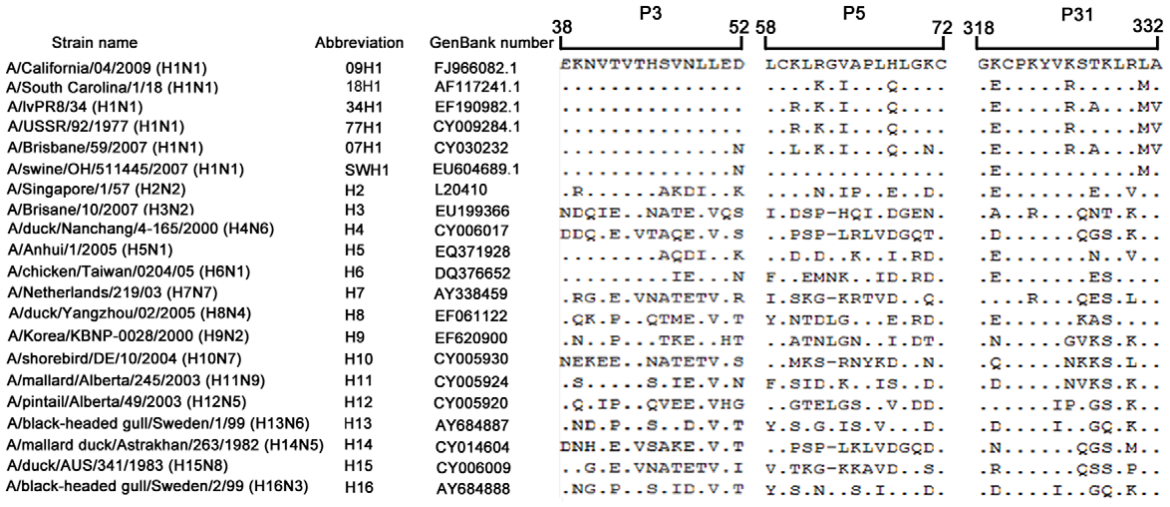
Alignment between the amino acid sequences of the peptides P3, P5, and P31 and the corresponding representative HA sequences of H1–H16 subtypes. Sequences of aa 38 to 52, 58 to 72, and 318 to 332 of the HA protein of A/California/04/2009 (GenBank number FJ966082.1) are aligned with the corresponding region of multiple HA proteins of H1–H16 subtypes and H1-subtype strains isolated in different years by BioEdit software.

To determine the conservation of the identified epitopes among IAVs, the aa sequences of P3, P5, and P31 were aligned with the corresponding aa sequences of all the 16 subtype HAs available in the GenBank. Fig. 4 is a representative of the alignment analysis, showing that P5 is identical to HA of H1 subtype strains. P3 is identical to HA of the H1 subtype, as well as highly identical to HA of the H2, H5, and H6 subtypes. These data are consistent with the specificity analysis by Western blot (Fig. 3A). Although anti-P31 antibody only recognizes the H1-subtype HA, it is similar to multiple subtypes.

To assess the identity levels of P5 and P31 sequences among the known IAV strains, *in silico* coverage analysis was performed. This analysis showed that the P5 peptide sequence could be identified in 91.6% (9860/10767) of the H1-subtype HA sequences available in the Influenza Research Database (http://www.fludb.org) (Table 2). Notably, this sequence scarcely presented (3.3% 792/23895) among the HAs of H2–H16 subtype IAVs. However, despite a high identity in the H1 HA proteins (93.1%), the peptide sequence of P31 also presented among the HAs of H2–H16 viruses (78.8%).

**Table 2:** Frequency of P5 and P31 epitope among H1 subtype HA.

Subtype	Query peptide	Number of hits/Total number of sequences	Coverage rate (%)*
H1	P5	9860/10767	91.6
H2–H16		790/23895	3.3
H1	P31	10027/10767	93.1
H2–H16		18830/23895	78.8

*Data by January 28, 2011.

Taken together, these findings indicate that the P5 peptide is H1-subtype specific and is conserved among H1 virus strains.

### Fine mapping of the epitope contained in P5

**Figure 5:**
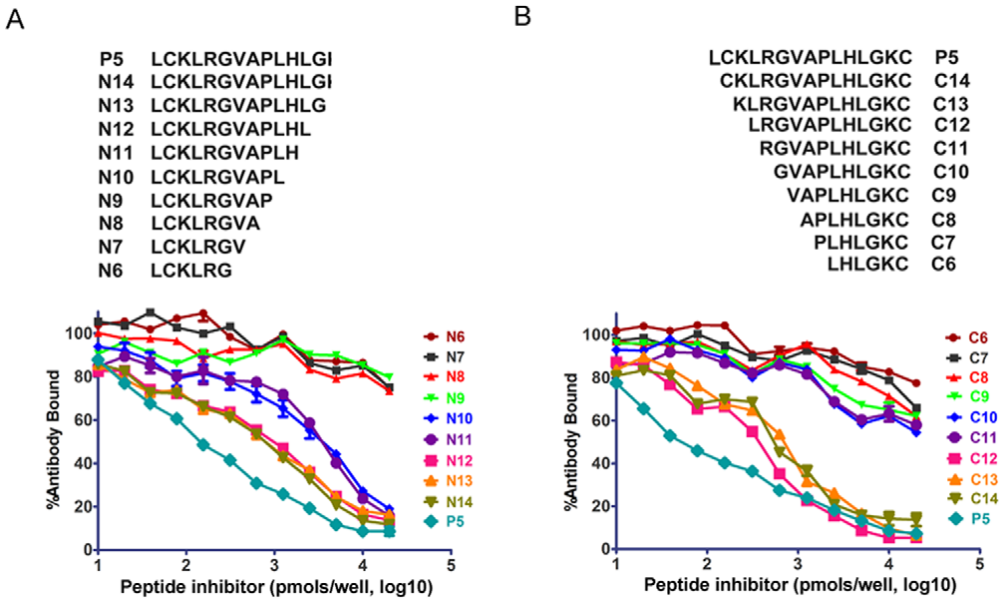
Inhibition of the binding of anti-P5 serum to parental peptide P5 by peptide homologs. Antiserum induced by the P5-KLH conjugate was tested by ELISA for its ability to bind to the P5 peptide in the presence of dilutions of peptides N6–N14 (panel A) or peptides C6–C14 (panel B).

**Figure 6:**
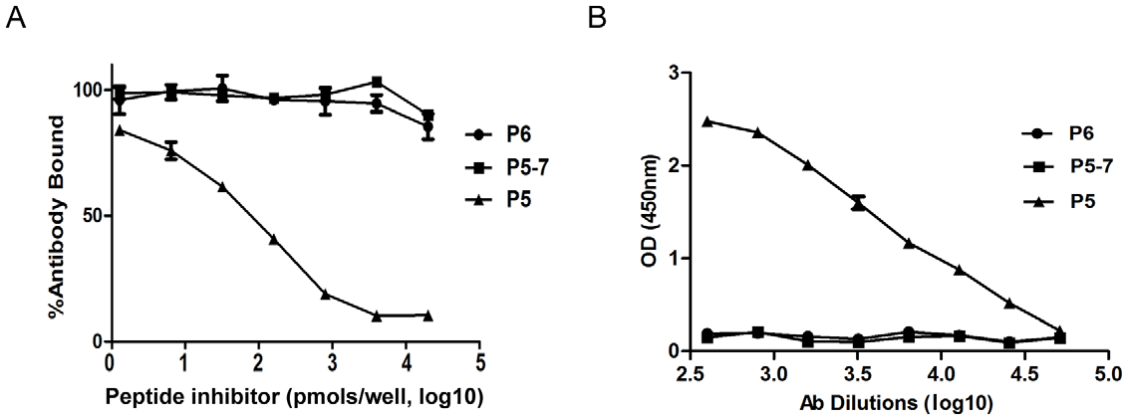
Peptide LRGVAPL fail to bind anti-P5 serum and inhibit P5-antibody interactions. (A) anti-P5 serum was tested by ELISA for its ability to bind to P5 peptide in the presence of dilutions of peptide P5–7 (LRGVAPL). P5 (LCKLRGVAPLHLGKC) and P6 (HLGKCNIAGWILGNP) were used as positive and negative controls, respectively. (B) The reactivities of anti-P5 sera (1∶400 dilution) with the peptides P6, P5–7, and P5 were analyzed by ELISA. The OD450nm values are expressed as mean ± SD.

To define the epitope contained in P5 precisely, a peptide-inhibition ELISA was performed. This experiment is reliable and is a standard methodology to determine the fine specificity of antigen-antibody reactions [31], [32]. A panel of short peptides derived from P5 (N6–N14, with C-terminus truncation; and C6–C14, with N-terminus truncation) were used to block the binding of anti-P5 antibody to coated P5. As shown in Fig. 5, antibody induced by the P5-KLH conjugate was inhibited by peptide N10–N14 and the parental peptide P5 to similar extents, whereas peptides N6–N9 only showed inefficient inhibition even at high molar concentrations. A similar pattern of inhibition was observed with the C-terminal conservative derivatives. Peptides C12, C13, C14, and the parent peptide P5 demonstrated comparable and efficient inhibition, whereas only slight inhibition was observed in peptides C6–C11 (Fig. 5B). Since the amino acid sequence LRGVAPL overlapped both peptides N10 and C12 (Fig. 5), we speculate that this sequence met the minimum requirements of binding to the anti-P5 antibody. However, the synthetic peptide LRGVAPL did not block the binding between P5 peptide and its antibody, nor did it directly bind to the P5 antibody (Fig. 6). As P5 exhibited the highest reactivities in the ELISA compared with the derived shorter peptides, we used P5 for further experimentations.

### Performance of a peptide-ELISA for H1 antibody detection

Inspired by the high specificity of P5 among the H1-subtype viruses, we developed an indirect ELISA assay using the P5 peptide to evaluate its performance as a diagnostic tool for H1 antibodies. The HI test was used as a reference method. As shown in Table 3, the overall agreement of these two methods was 87%, showing that the two methods have good correlation (Pearson correlation coefficient R = 0.741). The sensitivity and specificity of peptide-ELISA versus HI test was 96.5% and 74.4%, respectively, indicating the potential of the peptide-ELISA method in detecting antibody against H1-subtype IAVs.

**Table 3:** Comparison of peptide ELISA and HI test.

Peptide ELISA	HI test		Sum
	Positive	Negative	
Positive	55	11	66
Negative	2	32	34
Sum	57	43	100

χ^2^ = 51.81, P<0.01, Pearson correlation coefficient R = 0.741. The sensitivity and specificity of peptide-ELISA versus HI test was 96.5% and 74.4%, respectively. Calculation formula: sensitivity = TP/(TP+FN), specificity = TN/(FP+TN), where TP is the number of true positives, FN is the number of false negatives, FP is the number of false positives and TN is the number of true negatives [52].

## Discussion

In the present study, we identified immunodominant linear B cell epitopes on the H1N1pdm virus HA protein by a peptide scanning approach using H1N1pdm patients sera. We confirmed that an unidentified epitope was highly conserved among H1 subtypes viruses and showed a good correlation with results obtained using the HI test. These findings demonstrate the potential of epitope-based antibody detection in IAV diagnosis and surveillance.

IAV escapes the human immune system by continuous antigenic drifts and occasional antigenic shifts [33]. Attempts to develop universal vaccines and reliable diagnostic tools based on conserved epitopes of IAV are big challenges. Several epitopes that can elicit broad spectrum neutralizing antibodies have been identified recently. For example, Sui *et al.* identified a universal neutralizing epitope for group 1 HA [34]. Yoshida *et al.* reported a universal epitope in antigenic site B shared by H1, H2, H3, H5, H9, and H13 subtypes [35]. All these epitopes are conformation-dependent. In this study, we identified two epitopes (P5 and P31) which have not been identified previously (Fig. S1). The P5 (aa58–72) seems to be a dispatch that links the stem region and the globular region and is fully exposed on the surface, while P31 (aa 318–332d) is located in the middle of helix A and B on HA2. In contrast to previous studies, we found P5 to be a linear B cell epitope. Our data demonstrate that this epitope is highly conserved among H1 viruses (9860/10767, 91.6%). Because viral mutants that are resistant to conformational epitopes are more easily generated, the conserved linear epitope is more suitable for differentiating subtypes than conformational epitopes [33]. Hence, the epitope in P5 provides a new target for reliable diagnostics of H1-subtype IAVs.

Antigenic sites in IAV HA proteins of H1, H2, and H3 subtypes had previously been characterized by sequence analysis on antigenic variants and amino acid substitutions. These previously identified antigenic sites were mainly located in the globular head in the three-dimensional structure of the HA1 subunit of the HA molecule [36], [37], [38], [39]. For instance, five antigenic sites have been identified in HA of influenza virus A/PR/8/34, a well-known reference strain of H1N1 IAV [39]. Recently, several epitopes were identified in the HA2 unit [34], [40], [41]. Together with these reports, our results indicate that there are more epitopes than what we have imaged and the epitopes of IAV need to be further characterized.

The difference between our findings and previously identified epitopes can be explained by the difference of screening method used between our study and those of others. In previous studies, monoclonal antibodies from murine hybridoma cells were used to identify antigenic sites, while in this study we used a peptide scanning approach, which involves overlapping peptide library and human convalescent antisera—a strategy that is widely used for viral epitope identification [27], [42]. Given the fact that viral antigen can be recycled and presented as short peptides with different conformation during humoral immune response and these short peptides can be selected by B cell clones [43], and that convalescent sera from patients were much more complex than monoclonal antibodies from mice and can reflect the real immune responses during viral infection [44], our approach adds to the available techniques currently being used to identify linear epitopes in serologic tests.

Because HA pseudotyped lentivirus has been widely applied in the study on neutralizing antibodies against IAVs [30], we used this method to evaluate if the epitopes identified in this study could stimulate neutralizing antibodies. Our data showed that these epitopes could not elicit neutralizing antibodies in pseudovirion neutralizing assays due to their linear nature. Previous studies have shown that most neutralizing epitopes are conformation dependent [34], [35].

The length of B cell epitopes can vary from 5 to 20 amino acids [45], [46]. To map the epitope contained in P5, we performed a peptide-inhibition ELISA using a series of N-terminal and C-terminal truncated peptides. However, we found that the full-length P5 (15 aa in length) rather than truncated peptides showed strongest binding to the corresponding antibody (Fig. 5 and Fig. 6). As peptides N10 and C12 are the shortest truncated P5 that can bind to anti-P5 antibody and share a core sequence of LRGVAPL, we tested whether this sequence could be the epitope. However, the synthetic peptide LRGVAPL did not block P5-antibody interactions nor bind P5 antibody (Fig. 6). Thus, we speculate that adjacent amino acids to this sequence are also involved in the binding of antibody elicited by P5-KLH conjugates.

The concept of using linear epitopes in influenza virus diagnostics and control has not been extensively investigated. In a recent study, an epitope-blocking ELISA, which can universally detect antibodies to human H5N1 virus, has been developed [17]. Our results show that a peptide-ELISA based on the highly conserved H1 subtype-specific epitope can also be used for the detection of H1 antibodies, displaying good correlativity with the HI test. Our results indicate the potential of the P5 epitope in H1-subtype IAV diagnosis. However, the performance of this assay needs to be further evaluated in studies with large scale samples.

In conclusion, our data provide evidence that the H1 subtype HA harbors more epitopes than what has been found previously. The conservation of an epitope (P5, aa 58–72) in the H1-subtype HA of IAV and its near complete absence in other subtypes indicate that this epitope meets the critical requirements for diagnosis of H1 subtype influenza virus infections. The peptide-ELISA developed in our study may be applicable for sero-diagnosis and may serve as a universal diagnostic tool for H1-subtype IAV surveillance.

## Materials and Methods

### Synthetic peptides and their conjugates

To screen the H1-subtype specific epitopes, a set of 50 peptides spanning the amino acid sequences of the HA protein ectodomain of pandemic A/H1N1 2009 (H1N1pdm) influenza virus strain A/California/04/2009 were synthesized. Each peptide is 15 amino acids in length with five residues overlapping with the adjacent peptides [46] (Fig. 1). The peptides selected for immunization experiments are shown in Table 1. These peptides were conjugated with a carrier protein, the keyhole limpet hemocyanin (KLH; Sigma, St. Louis, MO), to improve their immunogenicity [47]. As the water solubility of peptides P5, P15, P16 and P31 were too low to conjugate with KLH directly, these peptides were first linked to 6-aminocaproic acid and then to the tripeptide KKC prior to being conjugated with KLH [48]. In addition, a family of short peptide homologs to the P5 peptide was also synthesized to fine map the epitope contained in the P5 peptide (Fig. 5). All of the peptides and their conjugates used in this study were synthesized by Sangon (Shanghai) Biotechnol Co., Ltd. (Shanghai, China). Each peptide was purified to homogeneity (>95% purity) by high-performance liquid chromatography and verified by mass spectrometry.

### Reference influenza virus strains

The reference influenza virus strains A/PR8/34 (H1N1) (abbreviated PR8), B/hubeiwujiagang/158/2009 (Yamagata lineage, abbreviated BY) and B/heilongjianghulan/116/2010 (Victoria lineage, abbreviated BV) were kindly provided by the Beijing Center for Disease Control and Prevention. The viruses were grown in MDCK cells as described elsewhere [5]. The titers of virus strains were determined by hemagglutination tests and expressed as hemagglutinating units (HAU). For Western blot analysis, the inactivated viruses were lyzed in a lysis buffer (50 mM Tris-HCl, 150 mM NaCl, 5 mM EDTA, 1% Triton-X, pH 7.4) supplemented with a protease inhibitor cocktail (Roche, Indianapolis, IN).

### Serum samples

Serum samples were collected from 11 convalescent patients during the early 2009 H1N1 pandemic in Beijing. The diagnostic criteria for H1N1 influenza virus infection of these patients fully followed the WHO's descriptions. Sera from 10 healthy blood donors were used as negative controls. In addition, serum samples collected from 100 blood donors were recruited to evaluate the peptide-ELISA assay developed in this study. All these samples were kindly provided by the Beijing Municipal Center for Disease Control and Prevention (Beijing, China) and written informed consent was obtained from all participants. All samples were coded prior to analysis to ensure anonymity and the procedures were approved by the Institutional Medical Ethic Review Board of the Institute of Pathogen Biology, Chinese Academy of Medical Sciences (Beijing, China).

### Expression of HA proteins

The full-length cDNA fragments corresponding to H2–H16 HA subtypes of IAV were inserted into the pCAGGS vector (purchased from Addgene) to express entire HA proteins (unpublished data). For H1 proteins, HA gene representing human IAV strains from different years (1918, 1934, 1977, 2007 and 2009) and a swine influenza virus strain were expressed by inserting the corresponding cDNA fragments into the pCAGGS vector in a similar manner. For the details of these influenza virus strains, please refer to Fig. 4. Recombinant plasmids were transfected into 293T cells (ATCC Number CRL-11268) using Lipofectamine 2000 (Invitrogen, Carlsbad, CA) according to the manufacturer's instructions. The cells were harvested and lyzed 72 hours after transfection. The expression of HA proteins was verified by Western blot analysis using murine antibodies against corresponding HA1 proteins (unpublished data).

### ELISA

The reactivities of the synthetic HA peptides or purified HA0 protein (eENZYME LLC, Montgomery Village, MD) with the convalescent-phase sera from H1N1pdm patients and the serum samples from mice immunized with peptide conjugates were determined by ELISA. Briefly, each peptide (1 µg/well) or protein (0.1 µg/well) was used to coat 96-well microtiter plates (Corning Costar, Acton, MA) in 0.1 M carbonate buffer (pH 9.6) at 4°C overnight. After blocking with 1% bovine serum albumen (BSA), the plates were incubated with indicated diluted serum samples (human or mouse) at 37°C for 2 hr, then washed four times with PBS containing 0.1% Tween 20 (PBS-T). Bound IgG antibodies were detected with horseradish peroxidase (HRP)-conjugated goat anti-human IgG or anti-mouse IgG (Sigma) at 37°C for 1 hr. After four washes with PBS-T, the reaction was visualized by addition of the substrate 3,3′,5,5′-tetramethylbenzidine (TMB, sigma). Color development was stopped by the addition of 50 µl/well of 2 M sulphuric acid after 15 min. The optical density at 450 nm (OD_450 nm_) was measured by an ELISA plate reader (Molecular Devices, Sunnyvale, California).

To evaluate the reactivity of the P5-derived short peptides with the anti-P5 antibody, peptide-inhibition ELISA assays were performed by adding dilutions of the peptides to a constant amount of the antibody elicited by the P5-KLH conjugates (1∶5000 dilution). Maximum binding to antigen-coated wells was observed in the absence of a peptide inhibitor. The antibody bound was expressed as a percentage of the maximum level of binding.

### Animal immunizations

Female BALB/c mice of 6–8 weeks old were immunized subcutaneously with various peptide-KLH conjugates mixed with Freund's Complete Adjuvant (Sigma) at 100 µg per injection. Boost injections were given at 2-week intervals with 50 µg antigen in Freund's Incomplete Adjuvant (Sigma) [49]. The antibodies were collected five days after the third boost. All the animal experiments were carried out in the facilities of the Institute of Laboratory Animal Sciences (ILAS), Chinese Academy of Medical Sciences (CAMS). All the experimental procedures were approved (permit number SLXKJ2009-0007) and supervised by the Animal Protection and Usage Committee of ILAS, CAMS.

### Western blot

At 72 hr post-transfection, the cells transfected with HA-expressing plasmids were harvested, pelleted, and lyzed in a lysis buffer (50 mM Tris-HCl, 150 mM NaCl, 5 mM EDTA, 1% Triton-X, pH 7.4) supplemented with a protease inhibitor cocktail (Roche, Indianapolis, IN). Aliquots of cell lysates (50 µg) or virus lysates were blotted after 10% SDS-PAGE onto nitrocellulose membranes (Pall, Port Washington, NY). The membranes were blocked with 5% non-fat milk and then incubated with the primary antibodies indicated in the figures at 4°C overnight. This was followed by incubation with the goat anti-mouse IRDye® Fluor 800-labeled IgG secondary antibody (1∶10, 000) (Li-Cor, Lincoln, NE). After washing, the membranes were scanned by the Odyssey® Infrared Imaging System (Li-Cor) and analyzed with Odyssey software. The molecular sizes of the developed proteins were determined by comparison with the pre-stained protein markers (Fermentas, Maryland, CA).

### Hemagglutination inhibition test (HI)

HI test was carried out as described elsewhere [5]. RDE treated serum samples were inactivated at 56°C for 30 min and two-fold serially diluted at an initial dilution of 1∶10. Twenty five µl of the diluted serum were incubated with 25 µl of the four hemagglutination units from reference influenza strains for 30 min at room temperature. The reference H1N1 IAV strains for HI test were A/Tianjinjinnan/15/2009(H1N1) and A/California/04/2009 (H1N1), respectively. 50 µl of 1% chicken erythrocyte suspension was added to each well and incubated for 30 min at 4°C. Positive reactions were recorded when the HI antibody titer was equal to or greater than 40.

### Production of pseudovirions and pseudotype neutralization test

H1N1pdm virus pseudotyped lentiviruses were produced in 293T cells co-transfected with pNL4.3-R^−^E^−^, HA and NA constructs using a polyethylenimine (PEI)-based transfection protocol [50]. Cell culture supernatants were collected 48 hr post-transfection, filtered through a 0.45 µm-pore size filter (Millipore, Billerica, MA ) and used in pseudotype neutralization test. Serum samples, heat inactivated at 56°C for 30 min, were diluted 40-fold in culture medium and mixed with an equal volume of diluted H1N1pdm influenza pseudovirions. After incubation at 37°C for 1 hr, 100 µl of pseudovirions (containing 50 ng/mL of HIV p24 gag protein) and serum mixtures were added into 96-well plates that contained 293T cells grown for 24 hr at initial 1×10^4^ cells. Infectivity was evaluated at 72 hr post-infection by luciferase assay. The percentage of infectivity of pseudovirions treated by tested serum to that of negative serum (as control) was calculated. 90% reduction in infectivity by serum addition is considered to be neutralizing activity [30]. Tests were run at least as duplicates.

### *In silico* coverage analysis

To assess the identity of the HA epitopes in IAVs, *in silico* analysis was performed by utilizing bioinformatics tools at the Influenza Research Database (http://www.fludb.org) [51]. The two programs used in this study were Search for Protein Sequences and Identify Short Peptides in flu proteins. The former program was used to define the number of total sequences in HA proteins according to the Subtype parameter (H1 or H2–H16). The latter program defined the number of hits (P5 or P31) in the H1 or H2–H16 total sequences. Because there are no standards for evaluating short peptide sequence homology, we chose the fuzzy match analysis to represent the identical level of a peptide sequence to HA proteins. The analysis type was chosen as fuzzy match, which meant >50% of characters were identical to the searched aa string. For example, entering GILGFVFTL may also find AILGFVFTI but not ALIGFVFSI.

### Statistical analysis

The Pearson correlation coefficient was calculated by Pearson chi square test for crosstab tables using SPSS software. The sensitivity and specificity of the peptide-ELISA versus HI test was determined by ROC curve analysis using SPSS software.

## Supporting Information

Figure S1 **Localization comparison between the identified peptides and the classical five antigenic sites in stereo view.** The HA monomer surface view of influenza virus A/PR/8/34 (PDB ID:1RU7) is shown and colored to illustrate the five antigenic sites (Sa, Sb, Ca1, Ca2, and Cb) and the identified peptides. From most membrane distal to proximal: P3 (blue), P31 (red), P5 (black), Cb (green), Ca1 (magenta), Ca2 (rainbow), Sa (yellow), and Sb (cyan).
(TIF)
