# Supplementary figures and images for: Identification of a Highly Conserved H1 Subtype-Specific Epitope with Diagnostic Potential in the Hemagglutinin Protein of Influenza A Virus
Source: PLoS One. 2011 Aug 19;6(8):e23374. doi: 10.1371/journal.pone.0023374 (PMC3158760; doi:10.1371/journal.pone.0023374)

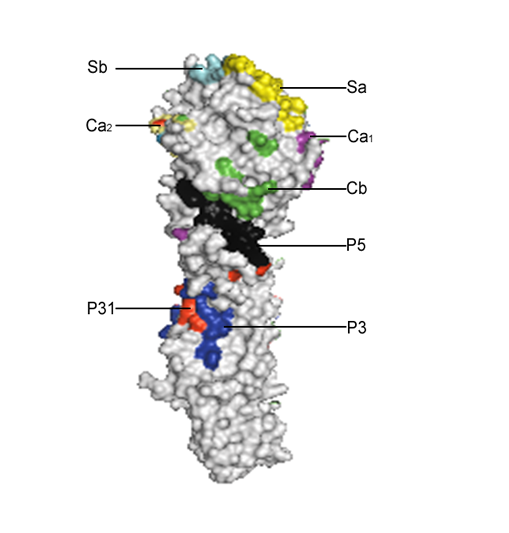

Supplement: Figure S1 — Localization comparison between the identified peptides and the classical five antigenic sites in stereo view. The HA monomer surface view of influenza virus A/PR/8/34 (PDB ID:1RU7) is shown and colored to illustrate the five antigenic sites (Sa, Sb, Ca1, Ca2, and Cb) and the identified peptides. From most membrane distal to proximal: P3 (blue), P31 (red), P5 (black), Cb (green), Ca1 (magenta), Ca2 (rainbow), Sa (yellow), and Sb (cyan). (TIF) [file pone.0023374.s001.tif]
